# Reducing mortality from childhood pneumonia and diarrhoea: The leading priority is also the greatest opportunity

**DOI:** 10.7189/jogh.03.010101

**Published:** 2013-06

**Authors:** Igor Rudan, Harish Nair, Ana Marušić, Harry Campbell

**Affiliations:** 1 Centre for Population Health Sciences and Global Health Academy, University of Edinburgh Medical School, Scotland, UK; 2 Croatian Centre for Global Health, University of Split School of Medicine, Split, Croatia; 3 Department of Research in Biomedicine and Health, University of Split School of Medicine, Split, Croatia

## Abstract

Pneumonia and diarrhoea have been the leading causes of global child mortality for many decades. The work of Child Health Epidemiology Reference Group (CHERG) has been pivotal in raising awareness that the UN's Millennium Development Goal 4 cannot be achieved without increased focus on preventing and treating the two diseases in low– and middle–income countries. Global Action Plan for Pneumonia (GAPP) and Diarrhoea Global Action Plan (DGAP) groups recently concluded that addressing childhood pneumonia and diarrhoea is not only the leading priority but also the greatest opportunity in global health today: scaling up of existing highly cost–effective interventions could prevent 95% of diarrhoea deaths and 67% of pneumonia deaths in children younger than 5 years by the year 2025. The cost of such effort was estimated at about US$ 6.7 billion.

Pneumonia and diarrhoea have been the leading causes of global child mortality for many decades [1]. Their relative importance in comparison to other causes of child deaths, such as malaria, preterm birth, birth asphyxia, accidents, neonatal infections and cancer, has become fully appreciated in 2003 through the work of the World Health Organization's and UNICEF's Child Health Epidemiology Reference Group (CHERG) [2]. The “Child Survival” series, published by *The Lancet*, has been pivotal in raising awareness that the UN’s Millennium Development Goal 4 cannot be achieved without increased focus on preventing and treating childhood infections – particularly pneumonia and diarrhoea – in low– and middle–income countries [3]. The next big step involved the “Global Action Plan for Pneumonia” (GAPP), which summarized the evidence on the epidemiology of acute lower respiratory infections in children, the key etiological agents, the main determinants of the disease, the available and emerging solutions and the main obstacles to their implementation [4]. GAPP’s landmark publication in 2008 showed that there are about 156 million new cases of pneumonia each year, and that about one in ten results in a severe episode that requires hospitalization, and a further 10% of severe episodes lead to deaths of affected children [5]. The paper also proposed that *Streptococcus pneumoniae* (SP), *Haemophilus influenzae* type B (Hib), respiratory syncytial virus (RSV) and influenza virus are the pathogens whose interplay is the most likely cause of the large majority of pneumonia deaths in children under five years of age [5]. This led to increased attention to pneumonia prevention through available vaccines against PC, Hib and influenza virus, to complement existing (antibiotic) case management strategies, including those delivered by community health workers. The underdeveloped and weak health systems in low–resource settings, where most deaths occur, cannot easily scale up antibiotic coverage to children who need them most, because of relatively low rates of access to health care [6]. Another obstacle to progress is infrequent care–seeking among parents who did not receive adequate education on this important health issue [6].

Four papers that followed the GAPP initiative, all of them published by *The Lancet*, have estimated the global and regional burden of SP [7], Hib [8], RSV [9] and influenza [10]. In parallel to understanding the burden of specific pathogens, new financial mechanisms have been developed – such as the Advance Market Commitment (AMC) – to reduce the prices of available vaccines and deliver them in low– and middle–income countries, where they would otherwise remain unaffordable to local governments [11]. The Global Alliance for Vaccines and Immunization (now called the GAVI Alliance) has been set up to raise funding to purchase these vaccines, with major contributions from the Bill and Melinda Gates Foundation who stood firmly behind the initiative to vaccinate children and prevent respiratory infections [12]. Those efforts ensured that most children in low–resource settings have received Hib vaccination by 2010, and pneumococcal vaccination coverage is also now being scaled up globally [13].

The similar effort for diarrhoea has been lagging behind until recently, when an international collaboration of researchers launched a “Diarrhoea Global Action Plan” (DGAP) [14]. Under the co–ordination of UNICEF, the World Health Organization, and USAID, the initiative has been merged with GAPP into “GAPPD” – “Global Action Plan for Pneumonia and Diarrhoea” [15]. This was a welcome move, because many risk factors are shared between the two diseases, and many approaches to control them could be delivered in parallel through an Integrated Management of Childhood Illness (IMCI) approach. Communities of researchers and policy–makers who are focused on diarrhoea control have also recently acquired the first vaccine effective in preventing an appreciable portion of the burden – rotavirus vaccine [16]. This vaccine will be added to GAVI portfolio to supplement PC and Hib vaccines for pneumonia [17].

The GAPPD group has recently been invited by *The Lancet* to write a series of papers that jointly address the epidemiology of childhood diarrhoea and pneumonia, the available cost–effective interventions, country–specific challenges and bottlenecks, and suggest policies that could accelerate progress in reduction of global mortality from the two diseases [15,18,19]. The series proposed that scaling up of existing highly cost–effective interventions could prevent 95% of diarrhoea deaths and 67% of pneumonia deaths in children younger than 5 years by the year 2025, if delivered at scale. The cost of this effort was estimated at about US$ 6.7 billion [15,18,19]. These activities, along with increased political stability in many low– and middle–income countries, their economic development, improved sanitation and access to care, progress in empowering and educating women in the society and strengthening health systems, could all contribute to very substantial reduction of the global burden of child mortality attributable to pneumonia and diarrhoea [18,20,21]. We should, therefore, conclude that addressing childhood pneumonia and diarrhoea is not only the leading priority, but also possibly the greatest opportunity in global health today.

There is a growing consensus that an improved understanding of the size of the burden, the leading risk factors and the relative contribution of the leading etiological causes; the available vaccines and other cost–effective interventions, such as community case management with antibiotics, oral rehydration sachets and zinc supplementation; and the momentum that many low– and middle–income countries have gathered in improving their economic outlook, have all provided the international health community with an unprecedented opportunity to substantially reduce the mortality from childhood pneumonia globally over the period of the next decade [15].

To support those ongoing initiatives, several leading medical journals published special issues, or series of articles, focused on childhood diarrhoea and pneumonia (and child survival in general) in the first half of 2013. As noted above, *The Lancet* will publish a four–paper series on childhood diarrhoea and pneumonia in April 2013, which is likely to have very large impact on the field [15,18,19]. *PLoS Medicine* published a *PloS Medicine* Collection on improving intervention delivery progress tracking and information systems in low–resource settings that could guide policy decisions. *BMC Public Health* is expected to publish a series of reviews that will present results of meta–analyses of the effectiveness of several key child health interventions, such as breastfeeding and vaccination, which should further strengthen the evidence base for the Lives Saved Tool (LiST) [22]. In this issue, *Journal of Global Health* joins this coordinated international effort launched by the CHERG group. We are bringing together several articles that should fill the remaining gaps in understanding acute respiratory infections and diarrhoeal disease in children. Rudan et al. revise national–level estimates of the morbidity, severe morbidity, mortality, etiology and risk factors for acute lower respiratory infections for 2010 [23]. Fischer–Walker et al. provide an assessment of co–morbidity between childhood pneumonia and diarrhoea [24]. Zwisler et al. use surveys to study the perception and use of oral rehydration sachets, antibiotics, and other therapies for diarrhoea in India and Kenya [25]; Wilson et al. analyse scaling up access to oral rehydration solution for diarrhea, learning from historical experience in low– and high–performing countries [26]; Simpson et al. add to this work by surveying caregivers in Kenya to assess perceptions of zinc as a treatment for diarrhea [27]. Finally, Zipursky et al. report on the global action plan for childhood diarrhea and show how research priorities were developed within this effort [14].
